# Autophagy in aging-related oral diseases

**DOI:** 10.3389/fendo.2022.903836

**Published:** 2022-08-05

**Authors:** Daniel Peña-Oyarzún, Carla San Martin, María Paz Hernández-Cáceres, Sergio Lavandero, Eugenia Morselli, Mauricio Budini, Patricia V. Burgos, Alfredo Criollo

**Affiliations:** ^1^ Physiology Department, Faculty of Biological Sciences, Pontificia Universidad Católica de Chile, Santiago, Chile; ^2^ Interdisciplinary Center for Research in Territorial Health of the Aconcagua Valley (CIISTe Aconcagua), School of Medicine, Faculty of Medicine, San Felipe Campus, Universidad de Valparaíso, San Felipe, Chile; ^3^ Instituto de Investigación en Ciencias Odontológicas (ICOD), Facultad de Odontología, Universidad de Chile, Santiago, Chile; ^4^ Advanced Center for Chronic Diseases (ACCDiS), Facultad de Ciencias Químicas y Farmacéuticas & Facultad de Medicina, Universidad de Chile, Santiago, Chile; ^5^ Departamento de Bioquímica y Biología Molecular, Facultad de Ciencias Químicas y Farmacéuticas, Universidad de Chile, Santiago, Chile; ^6^ Cardiology Division, Department of Internal Medicine, University of Texas Southwestern Medical Center, Dallas, TX, United States; ^7^ Department of Basic Sciences, Faculty of Medicine and Sciences, Universidad San Sebastián, Santiago de Chile, Chile; ^8^ Autophagy Research Center, Universidad de Chile, Santiago de Chile, Chile; ^9^ Centro de Biología Celular y Biomedicina (CEBICEM), Facultad de Medicina y Ciencia, Universidad San Sebastián, Santiago, Chile; ^10^ Centro de Envejecimiento y Regeneración (CARE-UC), Facultad de Ciencias Biológicas, Pontificia Universidad Católica, Santiago, Chile; ^11^ Centro Ciencia & Vida, Fundación Ciencia & Vida, Santiago, Chile

**Keywords:** autophagy, oral diseases, aging, periodontitis, oral cancer, periapical lesions

## Abstract

Autophagy is an intracellular degradation mechanism that allows recycling of organelles and macromolecules. Autophagic function increases metabolite availability modulating metabolic pathways, differentiation and cell survival. The oral environment is composed of several structures, including mineralized and soft tissues, which are formed by complex interactions between epithelial and mesenchymal cells. With aging, increased prevalence of oral diseases such as periodontitis, oral cancer and periapical lesions are observed in humans. These aging-related oral diseases are chronic conditions that alter the epithelial-mesenchymal homeostasis, disrupting the oral tissue architecture affecting the quality of life of the patients. Given that autophagy levels are reduced with age, the purpose of this review is to discuss the link between autophagy and age-related oral diseases.

## Introduction

**Figure 1:**
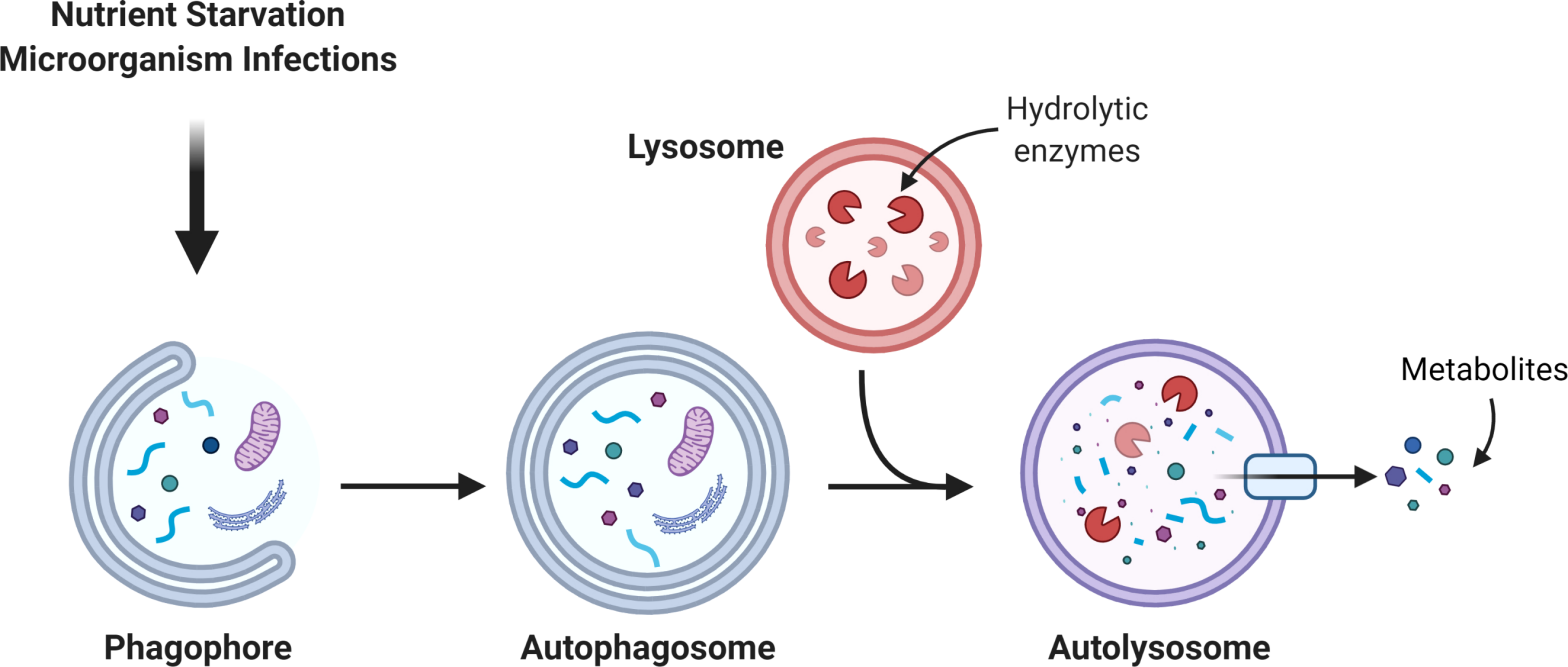
Autophagic pathway. The autophagosome is a double membrane organelle which sequesters intracellular material. Then, the autophagosome fuses with the lysosome to form the autolysosome where hydrolytic enzymes promote the degradation of the autolysosome cargo. The catabolism of the cargo generates simple new metabolites that turn back to the cytosol to be used in different metabolic pathways.

Macroautophagy (hereafter “autophagy”) is an intracellular degradation mechanism, evolutionarily conserved from yeast to mammals and present in basal conditions in all cells of the human body. During autophagy, intracellular organelles and macromolecules are engulfed in double membrane vesicles known as “autophagosomes”, which then fuse with a lysosome, to allow the recycling of the engulfed material (1). Cells undergo basal autophagy that recycles dysfunctional organelles and proteins, thereby maintaining cell homeostasis (2, 3). However, under stress conditions such as starvation or microorganism infections, autophagy may be upregulated to produce energy by catabolic degradation, or to remove the exogenous organisms (4, 5). This is critical for oral tissues, given the continuous exposure to bacteria and viruses in the oral cavity, the high metabolic requirement that allows the turnover of the oral mucosa cells and the constant physical stress that teeth are subjected to (6–8). Besides these pro-survival effects, autophagy is also referred to as type II programed cell death, meaning that when the pro-autophagic stimulus is extremely harsh, autophagy targets the whole cell for death (9). Therefore, the role of autophagy in oral diseases is very complex, and in many cases, depends on its levels and on the progression of the disease. An overview of autophagy is shown in **Figure 1**.

Aging is an irreversible biological phenomenon, affected by lifestyle, environment and genetics. The concept of aging goes beyond the concept of “lifespan”; it represents the different functional and anatomical changes in the tissues with time, which ultimately decreases the capacity to respond to internal and external stressors (10). As a homeostatic mechanism, autophagy is also affected by aging (11). A decreased autophagic tone is observed with age, while autophagy is enhanced during periodontitis, oral cancer, chronic oral infections and dental senescence (12). Studies on the physiological contribution of autophagy during aging of the oral tissues are scarce, however it has been reported that the dentin-secreting odontoblasts from old subjects (> 75 years old) show impaired fusion between autophagosomes and lysosomes, compared to those from young subjects (< 25 years old) (13). Additionally, autophagy is reduced in aged odontoblasts, which finally could affect cell homeostasis since odontoblasts are post-mitotic cells, highly dependent on cell homeostasis to promote tooth repair and healing (14). Similarly, autophagy is involved in other oral physiological responses, including tooth development and bacteria-host interaction (15). In this regard, the following question arises: Could the age-related decrease in autophagy explain the initiation and/or progression of age-dependent oral diseases, such as oral cancer, periodontitis and periapical lesions? Given that autophagy may promote either a pro- or an anti-survival effect on oral cells, and that autophagy has reciprocal control of over host immunity and energy expenditure, the answer to this question may not be as straightforward. Thus, the aim of this review is to discuss, the role of autophagy in human oral diseases associated with aging.

## Aging of the oral cavity

Aging is a natural process, in which the functional ability to cope with external and internal stressors is progressively reduced (10). Biological aging hallmarks include genome alterations (i.e. cumulative DNA damage, decreased Histone H3 methylation at Lys-9 and Lys-27, and increased Histone H4 acetylation at Lys-16 and Lys-20); a stable cell cycle arrest, also known as cellular senescence, caused by telomere attrition and increased expression of the cyclin dependent kinase inhibitor 2A CDKN2A/P16INK4A; and deregulated nutrient sensing pathways, associated to higher expression of the mechanistic Target of Rapamycin, MTOR, and augmented reactive oxygen species, ROS, production (16). Indeed, pharmacological inhibition of MTOR with rapamycin, which is also a well-known autophagy activator, dramatically increases lifespan in mice (17).

Oral structures may be divided into two: the teeth and the oral mucosa. Resembling an iceberg, the roots of the teeth are inserted in the alveolar bone below the gingival lining, leaving the dental crown visible at the surface (18). The structure of the teeth is formed by layers; the outermost layer is the enamel, a very hard mineral-based cover of hydroxyapatite of 2.5 mm wide which covers the dental crown (19). Enamel is produced by cells called ameloblasts during tooth development, after which they undergo apoptosis (19). The outermost layer under the gingival lining is called cementum. Cementum is composed of hydroxyapatite, collagen and proteoglycans, resulting in a much thinner and less hard structure (20). The cementum links the dental root to the alveolar bone by attaching the periodontal ligaments (21). The central layer of teeth is the dentin, a bone-like structure formed by projections of differentiated odontoblasts, which senses external stimuli like caries (22). Odontoblast bodies are concentrated in the inner layer of the teeth, the dental pulp, where nerves and blood supply are found (23). Finally, the oral mucosa is a stratified epithelium covering all the structures in the oral cavity, including the tongue. The cells that compose the oral mucosa are the keratinocytes, which produce cytokeratin and form an epithelial barrier that separates the oral cavity from the environment (24).

During oral aging, increased ROS levels provokes a reduction of organic matrix in the enamel, yielding a crystal structure that is even harder than younger enamel (25). This decreases the susceptibility to develop caries, but weakens the tooth in case of physical insults, leading to increased incidence of fractures and cracks (26). Thus, dentin undergoes sclerosis, because senescent odontoblasts deposit secondary dentin, thereby reducing the sensing capacity and dental pulp space (27). Histological analysis of aged odontoblasts shows that they also switch from a columnar to cuboidal arrangement while exhibiting accumulation of lipofuscin, a brown-yellow pigment that indicates decreased lysosomal digestion of lipids, as well as diminished mitochondrial oxidative function (28). Gingival retraction occurs during aging, therefore the cementum that normally lies below the gingival lining is progressively exposed (29). This implies that despite reduced susceptibility to caries, observed in aged enamel, increased incidence of caries at the root occurs with age, as the cementum has no resistance against the acid in the oral cavity (30). Compared to young individuals, aged dental pulp has decreased stem cell density and increased cellular senescence, caused by secondary dentin deposition and dystrophic calcification that blocks pulpal arteries (31). Besides gingival retraction, aged oral mucosa undergoes epithelial atrophy and increased subepithelial deposit of collagen, while reducing elastin content (32). This is observed histologically by decreased epithelial ridges, a wave-like epithelial arrangement in contact with the connective tissue where proliferative keratinocytes are found, which explains the slower regeneration of the oral mucosa in older individuals (33, 34).

## Aging-related oral diseases

### Oral cancer

Oral cavity cancer is a highly lethal disease, with a mortality rate of 50% after 5 years and an average of diagnosis of 62 years, affecting more men than women (2:1) (35). The prevalence of oral cancer is over 300,000 cases per year worldwide; it is the sixth most common type of cancer (36). The combination of tobacco and alcohol is by far the main risk factor, while other risk factors are vitamin deficiencies, particularly those of the B complex, and the human papilloma virus, HPV (37, 38). The main type of oral cancer is oral squamous cell carcinoma, OSCC, and other types of oral cancers account for less than 5% (39). OSCC is commonly located at the mobile tongue, in 20% of the cases, and the floor of the mouth in 30% of the cases (40). The proportion of oral cancers that proceed from leukoplakias ranges between 17% and 35%, highlighting the fact that dental consultation is crucial for early cancer detection (41). Indeed, precancerous lesions, defined by the WHO as “morphologically altered tissue in which cancer occurs more often than in normal autologous tissue”, also known as oral dysplasia, precede initiation of OSCC (41, 42). The clinical presentation is highly variable; ulcerated lesions are the most frequent, but in some cases bleeding, pain, or numbness may also be present (43).

### Periodontitis

Periodontitis is an extremely frequent disease, affecting nearly 70% of the global population (44). Periodontitis is a consequence of gingivitis, where a bacterial biofilm (dental plaque) forms on the gingival tissue, leading to inflammation and gingival retraction of the gum surrounding the tooth (45). In periodontitis, chronic inflammation and gingival retraction cause the migration of anaerobic gram negative bacteria such as *Porphyromonas gingivalis* and *Aggregatibacter actinomycetemcomitans* into the subgingival space (46). Since this continuous inflammation produces periodontal damage and alveolar bone resorption, diagnosis of periodontitis is commonly established when the probe depth of the gingival sulcus is over 3 mm (47, 48), which in severe cases can reach 6 mm (49). Prevalence of severe periodontitis is around 20% in adults between 35 and 44 years, while it is around 40% in adults over 60 years (50). Risk factors for periodontitis include smoking, diabetes mellitus, obesity, alcoholism, osteoporosis and stress (51). Infection with *P. gingivalis* disrupts oral epithelium arrangement and barrier function as proteolytic enzymes such as gingipains and collagenases are released (52). Also, during periodontal infections the antioxidant transcription factor NFE2 like bZIP transcription factor 2, NFE2L2/NRF-2, is severely downregulated, showing that oxidative stress is a key feature during periodontitis (53). Periodontal tissues show higher activation of nicotinamide adenine dinucleotide phosphate NADPH oxidase 4, NOX4, which catalyzes the production of superoxide anion after exposure with lipopolysaccharide, LPS, obtained from *P. gingivalis* (54). ROS like superoxide anion exhaust antioxidant catalase reserves, increasing progression of periodontal inflammation (55).

### Periapical lesions

Susceptibility to enamel fractures and secondary dentination increases with age, leading to pulp exposure to external stressors (26). Periapical lesions are necrosis of the dental pulp tissue associated with an exacerbated inflammatory response due to infections, also known as periapical granuloma (56). Periapical granuloma is characterized by the persistence of microorganisms in the radicular system of the dental pulp, most of them bacteria like *Acinetobacter johnsonii* and *Propionibacterium acnes*, or fungi such as *Candida albicans* (57, 58). Infiltration of macrophages, lymphocytes and plasmatic cells is followed by the production of a neutrophil-rich exudate, resulting in an acute oral inflammation (59). Chronic inflammation is established after the organism attempts to repair the damaged tissue by production of new odontoblasts, mesenchymal cells and matrix proteins, but being compromised by the presence of the microorganisms, forming a granuloma tissue (60). As a consequence of periapical granuloma, radicular cysts appear, spaces filled with extracellular liquid derived from epithelial fragments of the periodontal ligament after necrosis of the dental pulp (61).

## Mechanism of autophagy

**Figure 2:**
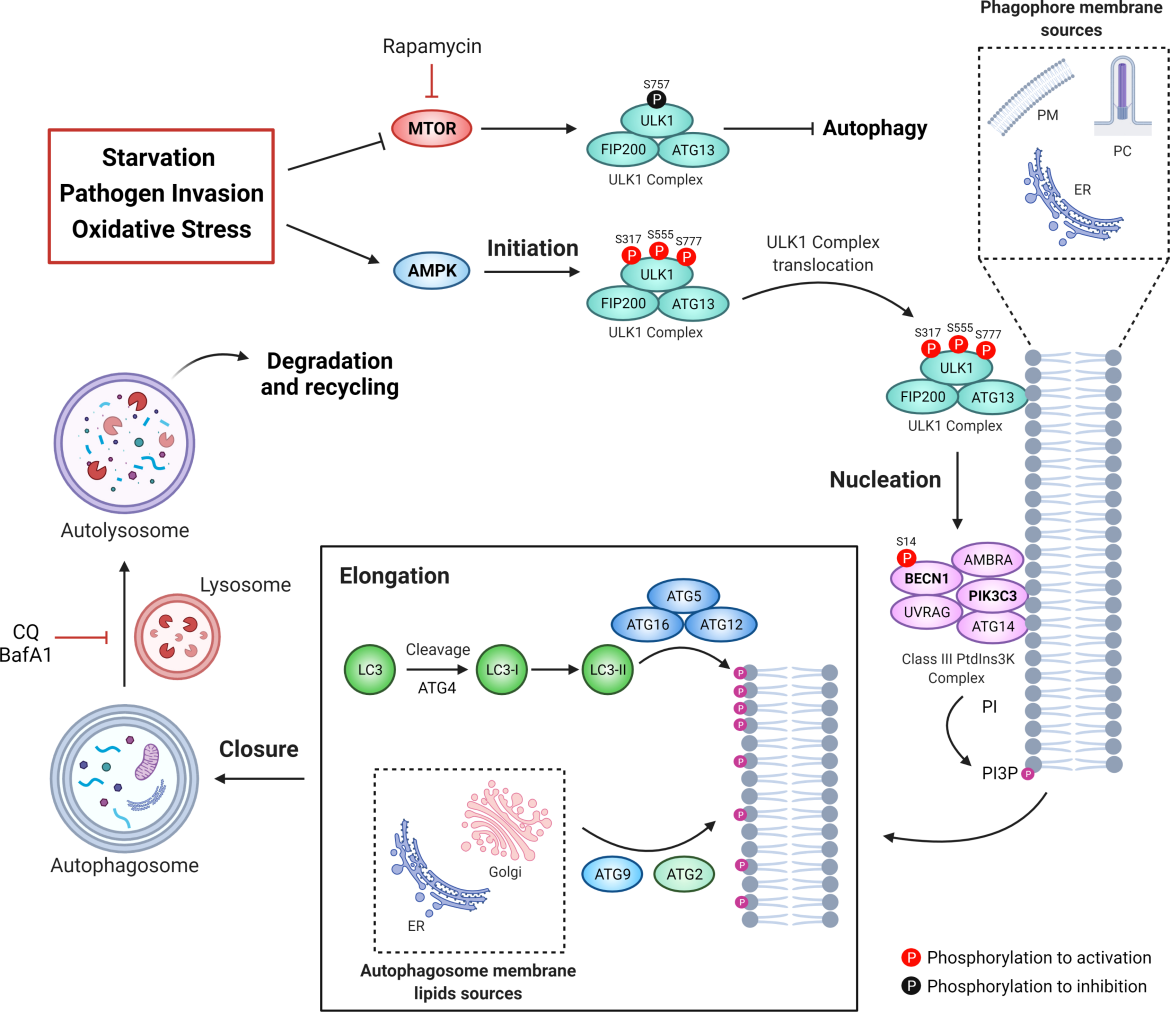
The autophagic machinery. Different types of stressors can be sensed by MTOR and AMPK. MTOR inhibits the ULK1 complex kinase activity phosphorylating its Ser757. Under stress conditions, AMPK activates ULK1 by phosphorylation on Ser317, Ser555, and Ser777, leading to the activation of the class III phosphatidylinositol 3-phosphate kinase complex (PtdIns3KC3). Then, active PtdIns3KC3 increases the levels of phosphatidylinositol 3-phosphate (PtdIns3P) in specific membrane micro domains, allowing the recruitment of proteins like the WD repeat domain, phosphoinositide interacting (WIPI). Thus, elongation of the phagophore membrane is mediated by two ubiquitination-like systems: the complex ATG12-ATG5-ATG16 and the conjugate LC3-phosphatidylethanolamine (PE), known as LC3-II. Additionally, both ATG2 and ATG9 participate in the elongation of the autophagosome through the trafficking of lipids from the endoplasmic reticulum or the Golgi apparatus. Once that the autophagosome membrane engulf intracellular components it encloses itself and then fuses with lysosomes to form the autolysosome. Chloroquine (CQ) or bafilomycin A1 (BafA1) can be used to block autophagosome-to-lysosome fusion, allowing the accumulation of autophagosomes.

Autophagy is a process in which cellular organelles called autophagosomes are formed to sequester and degrade intracellular material and macromolecules. Autophagy can be divided into five stages: initiation, nucleation, elongation, closure and fusion (62). The proteins that participate in the formation of autophagosomes are known as autophagy-related proteins or ATGs (63). In the initiation stage, signaling pathways like starvation, pathogen invasion, oxidative stress, among others, inhibit MTOR and/or activate AMP-dependent protein kinase, AMPK (64, 65). While AMPK-dependent phosphorylation activates unc-51 like autophagy activating kinase 1, ULK1/ATG1, MTOR-dependent phosphorylation of ULK1 inhibits it (66). Active ULK1 traffics to endomembrane domains, where it phosphorylates the protein Beclin 1 (BECN1) at Ser-14 (67). BECN1 allows the formation of the class III phosphatidylinositol 3-phosphate kinase, PtdIns3KC3, which phosphorylates the phosphatidylinositol lipids, creating “nucleation” signals for the recruitment of other ATGs (68, 69). Elongation of the autophagosome requires the incorporation of the Microtubule Associated Protein 1 Light Chain 3, MAP1LC3, (or just LC3) into the autophagosome membrane (70), which is previously cleaved by the ATG4 protease and conjugated with phosphatidylethanolamine by the ATG5-ATG12-ATG16 complex to forming LC3-II (71, 72). Measurement of the LC3-I to LC3-II conversion or quantification of LC3-II levels are common strategies to study autophagy (73). Other ATGs proteins such as ATG2 and ATG9 are involved in the trafficking of lipids allowing expansion of the autophagosome (74). Finally, the autophagosome membrane encloses and fuses with a lysosome that contains hydrolytic enzymes and low pH, to allow cargo degradation (75). Therefore, autophagic flux, known as the complete process beginning with autophagosome formation and degradation of the enclosed material, is usually evaluated by the use of autophagosome-lysosome fusion inhibitors (i.e. chloroquine or bafilomycin-A1) or by the tracking of the adaptor protein SQSTM1/p62, which targets poly-ubiquitinated proteins to LC3 on the autophagosome, allowing their degradation (76). Indeed, lower levels of SQSTM1/p62, reflect higher autophagic flux (77, 78). The mechanism of autophagy is depicted in **Figure 2**.

## Autophagy and age-related oral diseases

Reduced autophagy is observed in almost all tissues during aging, including oral cells. For instance, odontoblasts from 75 year-old individuals show accumulation of autophagic vesicles compared to odontoblasts from 25 year-old individuals (13). In contrast to younger odontoblasts, older odontoblasts display higher co-localization of mitochondria and lysosomes, accompanied by accumulation of lypofusin, suggesting lysosomal dysfunction (13). Thus, autophagic vesicles accumulation in older individuals is a result of decreased autophagy flux. In fact, reestablishing autophagy with rapamycin, attenuates aged-induced periodontal bone loss and gingival inflammation in mice, suggesting that autophagy-based pharmacological treatment could delay oral aging (79). In the next sections, we will describe the contribution of autophagy in age-related oral diseases.

### Oral cancer

The role of autophagy in most cancers is complex and controversial. Indeed, autophagy has been extensively described as a “double-edged sword”, with different roles during carcinogenesis and cancer progression (80). Despite oral cancer has been proposed to follow this double-edged sword behavior (81), some considerations need to be addressed when oral and non-oral cancers are compared.

Previous work shows that both heterozygous deletion of ATG5 and specific homozygous deletion of hepatic ATG7 in mice, result in hepatomegaly and liver tumor formation, respectively, in 6-month-old mice (82). These tumors accumulate SQSTM1/p62 and ubiquitin aggregates, as well as 8-hydroxydeoxyguanosine, 8-OHdG, suggesting that reduced autophagic degradation is associated with oxidative stress (82). Similarly, spontaneous neoplastic formations in lung and liver have been found in mice with a heterologous deletion of BECN1 (83, 84), implying that impaired autophagy promotes carcinogenesis in non-oral tissues. In oral carcinogenesis, tumor xenografts in mice obtained by subcutaneous injection of TSCC (human tongue squamous cell carcinoma) cells downregulated for BECN1 display significant increase in volume and weight, compared to control TSCC xenografts (85). Additionally, in mice treated for 16 weeks with 4-nitroquinoline *N*-oxide, 4-NQO, a cigarette-smoke compound (86), progression of the oral mucosa from normal to dysplastic, and then from dysplastic to cancerous positively correlates with the increase in LC3 and SQSTM1/p62 levels, suggesting that the malignant transformation of normal oral cells is associated with decreased autophagy (87). Altogether these studies indicate that both in oral and non-oral tissues, inhibition of autophagy is involved in cancer initiation.

During progression of non-oral cancers, cancer cells increase their autophagic tone to overcome the stress of crowding, hypoxia and nutrient deprivation. For instance, it has been demonstrated that deletion of scribble/scrib (the ortholog of human scribble planar cell polarity protein, SCRIB) in *Drosophila melanogaster* (scrib KO flies), a well-known model of eye tumor that invades the central nervous system, reduces lipid droplet content in the adipose tissue and increases muscle atrophy measured by micro-computerized tomography, as well as increases LC3 processing in all the aforementioned tissues, suggesting that scrib-deficient tumor cells obtain nutrients by wasting host organs (88). Autophagy is required for this systemic organ wasting, as scrib KO flies that do not express ATG13, part of the ULK1 kinase complex, show reduced muscle atrophy and increased lipid droplet content, compared to scrib KO flies (88). Similarly, treatment of pancreatic adenocarcinoma xenografts *in vitro* with the inhibitor of the autophagic flux chloroquine, a chemical compound that blocks the fusion between lysosome and autophagosome, decreases oxygen consumption and impairs tumor growth (89). Consistently, treatment of MCF7 breast cancer xenografts with chloroquine reduces tumor viability (90). Given that these cells display higher levels of LC3-II and autophagosome vesicles under serum deprivation, authors conclude that autophagy is induced to support tumor growth in conditions of starvation (90).

On the other hand, progression of oral cancer should be interpreted with caution, or at least better dissected in a specific time frame. Indeed, the treatment of Cal 27, a human OSCC cell line, with up to 2 mM melatonin, increases LC3-I to LC3-II conversion and reduces SQSTM1/p62 levels, while increasing Caspase 3 cleavage, indicating that melatonin treatment induces both autophagy and apoptosis (91). Additionally, Cal 27 subcutaneous tumor xenografts in mice reduce their weight after treatment with 100 mg/kg of melatonin, suggesting that induction of autophagy promotes apoptosis in OSCC cells (91). In this model, autophagy is induced through the transcription factor binding to IGHM enhancer 3, TFE3, which upregulates the expression of autophagy-related genes, such as *atg7* and *lamp1* (92). Similarly, treatment of Cal 27 cells with sepantronium bromide, a chemical inhibitor of Survivin, not only leads to apoptosis, but also triggers autophagy, as it has been observed by the increase in LC3 lipidation and SQSTM1/p62 degradation (93, 94). Autophagy is upregulated in this model due to decreased MTOR activity, as reduced of MTOR auto-activating phosphorylation at Ser-2448, as well as diminished phosphorylation of ribosomal protein S6, RPS6, at Ser-235 and Ser-236, a downstream target of MTOR (93). Most importantly, tamoxifen-induced double knock-out mice for transforming growth factor β1 receptor, TGFBR1, and phosphatase and tensin homolog, PTEN, display a reduction in tumor growth, as well as a weak immunohistochemical staining for MTOR phosphorylation at Ser-2448 and SQSTM1/p62, after exposure with 5 mg/kg sepantronium bromide (93). All together, these findings indicate that induction of autophagy during oral cancer progression negatively affects tumor growth.

Other studies have shown that biopsies from patients with poorly differentiated OSCC show higher immunohistochemical staining against LC3, BECN1 and SQSTM1/p62, indicative of an impaired autophagy (95–97). However, given that increased levels of ATG5 and ATG9, and therefore higher autophagy, correlates to unfavorable overall survival of cancer patients (98), it is possible that oral cancer progression promoted by autophagy inhibition occurs only during advanced stages of OSCC, while in the early stages of oral cancer development autophagy is upregulated to support tumor progression. Wound healing and transwell assays in TSCC cells treated with rapamycin show reduced cell migration and invasion when compared to control cells, suggesting autophagy inhibition in the later stages of cancer progression promotes cell migration and invasion (99).

The change in the “autophagic behavior” of OSCC may be explained by the cancer microenvironment, particularly by the carcinoma-associated fibroblasts, CAFs (100). CAFs release interleukin-33, IL33, and chemokine (C-C motif) ligand 7, CCL7 inhibiting autophagy in OSCC cells and promoting proliferation and epithelial-mesenchymal transition, EMT (101, 102). CAFs also transfer their mitochondria to the OSCC cells and inhibit AMPK, explaining the metabolic switch of the OSCC cells to lactate production, their resistance to metformin treatment and the impairment of autophagy (103, 104). This is interesting because, given that oral autophagic status is decreased with age, OSCC cells may be more prone to acquire malignant traits on their own than from interacting with the oral mesenchymal cells. This also suggests that autophagy-based treatments for oral cancer should consider the age of the patients.

### Periodontitis

During periodontitis, pathogens like *P. gingivalis* use the autophagic machinery of oral keratinocytes, dendritic cells and macrophages to survive and disseminate (105). After entering myeloid dendritic cells, *P. gingivalis* is transported within early endosomes, as indicated by co-localization between RAB5A positive vesicles and *P. gingivalis* (106). Inside the eukaryotic cell, several virulence factors of *P. gingivalis* increase LC3-II and BECN1 levels, while reducing caspase activation and annexin V staining, indicating that periodontal pathogens induce autophagosome formation and suppress apoptosis (107). For instance, the penta-acylated form of LPS from *P. gingivalis* not only increases LC3 vesicle formation, but also quadruples autophagosomes diameter, compared to the tetra-acylated form (108). Given that, contrary to the tetra-acylated LPS from *P. gingivalis*, the penta-acylated form is recognized by the toll-like receptor 4, TLR4, interaction of pathogen associated molecular patters, PAMP, with TLR molecules is crucial for *P. gingivalis*-dependent autophagosome formation (108). In addition, this penta-acylated LPS from *P. gingivalis* reduced melanoregulin, MREG, levels, a protein required for lysosomal hydrolase activity of Cathepsin D and β-N-Acetylglucosaminidase, suggesting that *P. gingivalis* PAMPs diminish lysosomal activity (108, 109). Similarly, the mannose content of the fimbria major subunit Mfa1, mfa1, of *P. gingivalis* is recognized by the C-type lectin receptor CD209/DC-SIGN inside the oral mucosa cells, ultimately downregulating the expression of lysosomal associated membrane protein 1, *Lamp1* (106). *P. gingivalis* remains hidden inside the autophagosomes, from where it obtains the nutrients to its replication, but blocks the fusion with the lysosome, avoiding degradation (106, 110). This is relevant since, in the literature, increased autophagosome formation by *P. gingivalis* is commonly confused with reduced autophagy flux. In fact, recovering autophagy in *P. gingivalis*-infected macrophages with calcitriol, 1α, 25-dihydroxyvitamin D3, decreases the survival of bacteria while increasing lysosomal function (111). Similar results are observed after stimulation of *P. gingivalis*-infected myeloid dendritic cells with rapamycin (106). Nevertheless, butyrate, a short chain fatty acid produced by anaerobic bacteria of the dental plaque, induces autophagy, promoting caspase-independent cell death in oral keratinocytes (112). This suggests that some metabolic byproducts of the periodontal pathogens may increase autophagy in oral gingival tissues, while other structural components of periodontitis-related pathogens are important for increasing autophagosome formation but then impair the autophagic flux. Despite the contribution of the autophagosome to *P. gingivalis* survival within the intracellular eukaryotic microenvironment, not all *P. gingivalis* that enter through early endosomes are targeted by the autophagic system, as some bacteria will be routed to the recycling endosome pathway to allow exocytosis. Indeed, gingival epithelial cells knocked-down for recycling endosome marker RAB11, the small GTPase required for interaction with the exocyst components RAS like proto-oncogene A, RALA, or the exocyst complex components 2/3/84, EXOC2/3/84 and then infected with *P. gingivalis*, display decreased colony formation units, cfu, in agar when the extracellular culture medium of the cells is plated (113). Consistently, in the same experimental conditions, increased bacterial cfu is observed when plating the intracellular content of the infected gingival cells, suggesting impaired exocytosis (113). Given that RALB participates during starvation-induced formation of the autophagosome by assembling BECN1 components (114), it is possible that *P. gingivalis* proliferates within the autophagosome and then escapes to infect neighboring gingival cells using the exocyst pathway. However, this has not been proven yet.

On the other hand, the interaction between oral pathogens and oral host cells is modulated by aging. A study where the 16S rRNA of the oral microbiome from “clinically healthy” old (> 40 years) or young subjects (around 23 years), as well as from “clinically confirmed periodontitis” patients, was isolated and sequenced, showed that the oral microbiome from old (but healthy) individuals is an intermediate condition between healthy young subjects and periodontitis patients (115). Indeed, higher levels of *P. gingivalis*, *Tannerella forsythia* and *Treponema denticola*, together known as the “red complex pathogens,” involved in the development of chronic periodontitis, increased 48, 25 and 55 fold, respectively in old healthy subjects when compared to young healthy persons (115). It has been previously suggested that this increased prevalence in red complex pathogens with age is due to a low-grade pro-inflammatory condition or “inflammaging” (116). In fact, assessment of the gingival fluid from aged individuals (> 65 years) and young individuals (< 25 years), both groups without periodontitis, show augmented B cell infiltration and IgG3 levels, suggesting an immune shift towards chronic inflammation (117). The relation between autophagy and inflammaging has been comprehensively reviewed elsewhere (118). Briefly, autophagy recycles aged mitochondria, thus preventing the rise of ROS levels and blunting the activation of the danger sensor NLR family pyrin domain containing 3, NLRP3, a protein that mediates IL1B release through caspase 1 activation (118). Whether autophagy predisposes the development of chronic periodontitis in old subjects by reducing inflammaging is not known yet.

It is important to understand that increased ROS levels does not necessarily leads to periodontitis, since the physiologic response following bacterial infection of the oral mucosa and periodontal tissue is by oxidative stress-driven autophagy (119). Indeed, neutrophils contribute to ROS formation upon pathogen infection in a mechanism that is associated with higher expression of ATG12 and LC3 (120). Because treatment of periodontitis-related neutrophils with N-acetylcysteine avoids the gene expression of ATG12 and LC3, autophagy is actually induced by ROS in these cells, possibly aiding cell survival (120). This could be important to overcome the downregulation of NFE2L2 observed in periodontitis (53). As oral autophagy is decreased with age, increased periodontal toxicity by oxidative stress is observed, resulting in elevated periodontitis-induced bone resorption (121, 122).

### Periapical lesions

Autophagy is crucial for odontogenesis, since it provides energy and removal of wasted intracellular components in enamel and oral epithelial cells (123). Compared to dental pulp cells from young rats, senescent dental pulp cells obtained from old rats display increased immunohistochemical staining of LC3 and BECN1, accompanied by decreased levels of peroxisome proliferator activated receptor gamma PPARγ (124), a transcriptional factor required to induce the expression of autophagy related proteins (125, 126). In this model, stimulation with LPS exhausts autophagy related proteins, suggesting that accumulation of LC3 and BECN1 is a consequence of an impaired autophagy flux (124). Indeed, adenoviral upregulation of PPARγ recovers autophagosome formation to cope with LPS stimulation (124). Additionally, dental pulp stem cells obtained from canine and human tooth root treated with C-X-C motif chemokine ligand 12, CXCL12, show increased levels of LC3-II and ATG5-ATG12 complex, as well as inhibition of the MTOR signaling, indicating that CXCL12 promotes autophagy in dental pulp stem cells (127). Authors demonstrated that CXCL12-dependent autophagy is required for stem cell migration as CXCL12 increased pore migration in transwell assay, but the phenomenon was prevented by treatment with the autophagic inhibitor chloroquine (127). Thus, in this model, high expression of ATG5-ATG12 complex and LC3, indicative of increased autophagy, is critical for development and regeneration of the dental pulp (128). Therefore, in old mice, autophagosomes are accumulated as a result of decreased autophagy flux, reducing periapical lesion repair. For instance, a rapid rise in LC3-II/LC3-I ratio, ATG12 levels and activating phosphorylations of ULK1 occur in murine odontoblasts after treatment with hydrogen peroxide, showing that increased autophagy is required for early dental repair (129). In the same model, treatment with simvastatin, an MTOR inhibitor of the statin-family drug for treatment of obesity, reduced dental destruction (129). Increased expression of ATG5-ATG12 and activating phosphorylation of AMPK, accompanied by augmented levels of hypoxia inducible factor 1 subunit alpha, HIF1α, has been observed in periapical granulomas (130). Given that the treatment of pre-odontoblast cell line mDPC6T with chloroquine reduces cell viability in presence of LPS, we can conclude that autophagy in periapical lesions promotes survival of odontoblasts under harmful stimulus (131). Therefore, as autophagy flux is reduced with age, dental repair capacity will be progressively limited, shifting the focus towards the search for novel polymers with better repair capacity and integration in older individuals (132).

**Figure 3:**
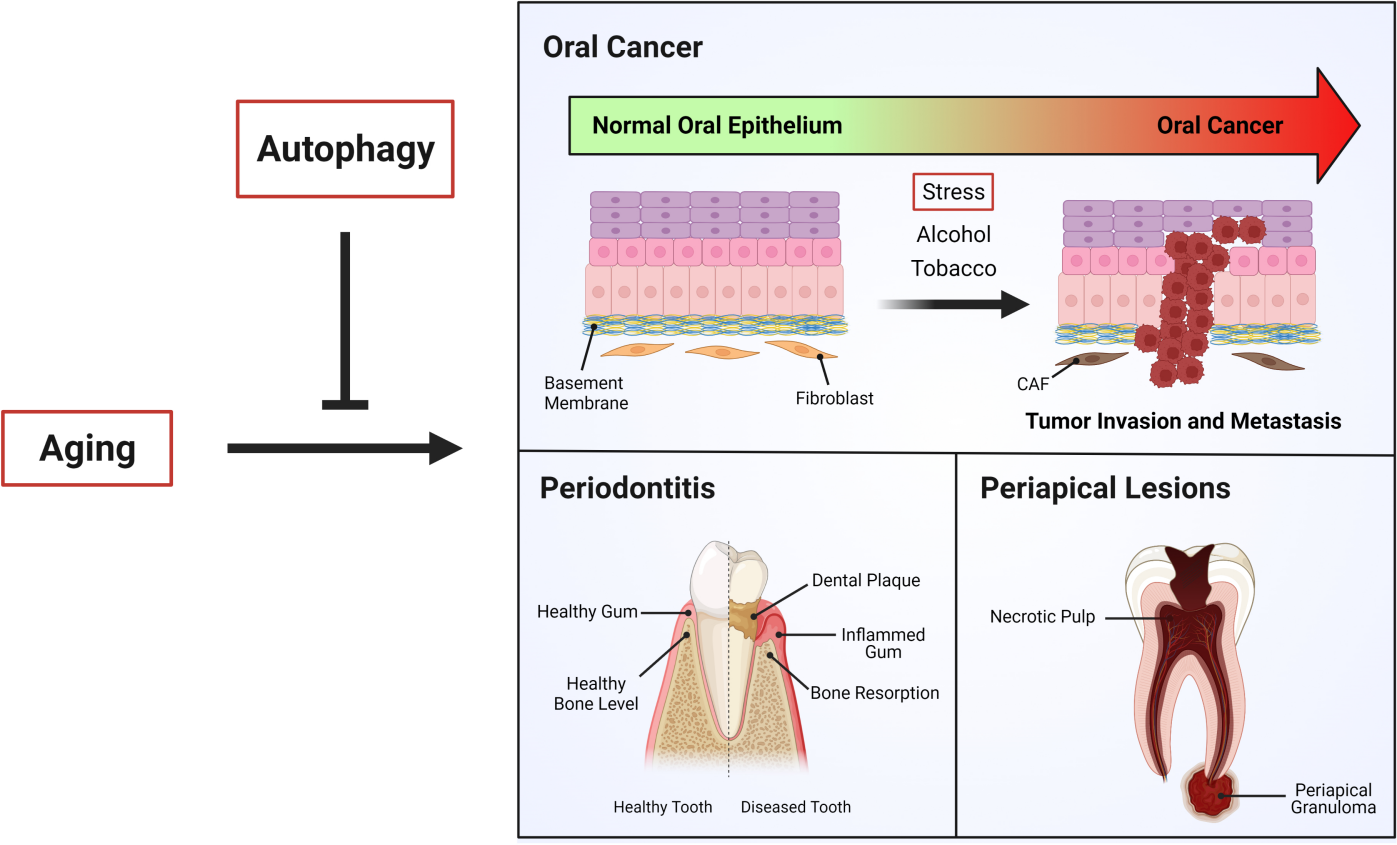
Autophagy and age-related oral diseases. Aging is a physiological process in which different cellular mechanism decline, leading in most of cases age-related diseases. “Autophagy”, which is impaired during aging, has been shown be critical to the control of age-related oral diseases such as oral cancer, periodontitis and periapical lesions.

Interestingly, eukaryotic pathogens like *C. albicans* associated with periapical lesions require autophagy for infection and virulence. Fungal expression of ATG9 has been shown to allow intracellular trafficking (133). Thus, VPS34, the fungal homologue of PIK3C3, forms a complex with the Vma7 subunit of H^+^-ATPase, contributing to vacuolar acidification and thereby increased autophagy-lysosome pathway activation (134). Additionally, *C. albicans* strains with deletion of VPS34 undergo cell death in stress conditions, such as during nitrogen starvation (134). Indeed, their survival is severely impaired in this condition, indicating that fungal autophagy is key for *C. albicans* virulence (134). This means that autophagy is increased in *C. albicans* during periapical lesions, but decreased in older individuals that are being infected. The role of autophagy in age-related oral diseases is summarized in **Figure 3**.

## Concluding remarks

Aging is a natural and irreversible process that all of us are experiencing. The complex interplay of the molecular mechanisms that are altered during aging finally leads to reduced sensitivity and adjustment capacity against external and internal changes in the cells. One of the main tools that cells have to control energy balance and recycling of wasted molecules is autophagy. Autophagy, as a manifestation of this reduced homeostatic capacity, begins to diminish with age. Susceptibility to certain pathologies also increases, especially those associated with the oral cavity, which is in constant stress by interaction with the environment, like oral cancer, periodontitis and periapical lesions. What is important to note is that age-dependent autophagic decrease is in most cases a result of impaired fusion between autophagosomes and lysosomes; this implies that autophagosome formation may actually occur, which for example favors the survival of *P. gingivalis* and *C. albicans* during periodontitis and periapical lesions. It seems that progression of aged-associated oral diseases is explained both by reduced lysosomal activity and by the accumulation of autophagosomes that protect foreign pathogens. The role of autophagy in oral cancer is difficult to establish, as it depends on whether we are observing the initiation, the progression, or the late development of the cancer. The orchestrated shift in autophagy in oral cancer cells may be defined by the tumor microenvironment in a specific temporal manner.

## Author contributions

Writing: DP-O, CM, SL, EM, PB, AC Design of Figures: MH-C, AC. Writing, Corrections and Editing: MB. All authors contributed to the article and approved the submitted version.

## Funding

This work was supported by the Agencia Nacional de Investigación y Desarrollo de Chile (ANID, Chile): FONDECYT [1200499 to EM and 1211329 to AC]; PIA-ANID [ACT172066 to EM, MB, PB and AC]; FONDAP [15130011 to SL and AC]; FONDECYT Post-doctoral fellowship [3200313 to DP-O and 3210630 to MH-C]; ANID/BASAL/FB210008 and ANID/BASAL/ACE2100099 to PB. Programa de Movilidad Internacional Santander Universidades, Versión 2021 to AC

## Acknowledgments

We sincerely thank everyone in the Criollo laboratories for discussion and constructive criticism. Figures made in BioRender.com.

## Conflict of interest

The authors declare that the research was conducted in the absence of any commercial or financial relationships that could be construed as a potential conflict of interest.

## Publisher’s note

All claims expressed in this article are solely those of the authors and do not necessarily represent those of their affiliated organizations, or those of the publisher, the editors and the reviewers. Any product that may be evaluated in this article, or claim that may be made by its manufacturer, is not guaranteed or endorsed by the publisher.
